# A comparison of foot posture and walking performance in patients with mild, moderate, and severe adolescent idiopathic scoliosis

**DOI:** 10.1371/journal.pone.0251592

**Published:** 2021-05-17

**Authors:** Feilong Zhu, Qianqin Hong, Xiaoqi Guo, Dan Wang, Jie Chen, Qian Zhu, Chong Zhang, Wei Chen, Ming Zhang

**Affiliations:** 1 The Affiliated Xuzhou Rehabilitation Hospital of Xuzhou Medical University, Xuzhou Rehabilitation Hospital, Xuzhou, China; 2 Department of Rehabilitation Medicine, Xuzhou Central Hospital, The Xuzhou Clinical College of Xuzhou Medical University, Xuzhou, China; West Park Healthcare Centre, CANADA

## Abstract

**Background:**

Adolescent idiopathic scoliosis (AIS) is the most common form of scoliosis. However, the underlying mechanisms linking spinal curvature in AIS to foot characteristics and walking performance remain unclear.

**Objective:**

This study aimed to compare walking performance between adolescents with mild, moderate, and severe scoliosis and matched healthy peers with foot posture as covariates.

**Methods:**

This cross-sectional study of 96 adolescents was conducted between April 2020 to October 2020 in China, with 32 healthy peers in the control group and 64 patients in the AIS group. Foot posture and morphology, plantar pressure distribution, and gait characteristics were analyzed. One-way analysis of variance with Bonferroni correction and a post hoc comparison of the mean differences between the different groups was performed. Multiple analyses of covariance adjusted for age, sex, body mass index, foot posture index (FPI), arch index (AI), and walking speed were performed.

**Results:**

Of the 64 adolescents with scoliosis, 18 had mild AIS, 32 had moderate AIS, and 14 had severe AIS. The AI and FPI were much higher in the moderate and severe AIS groups (p = 0.018) and the severe AIS group (p<0.001), respectively, than in the control group. The severe AIS group had advanced and longer midstance (p = 0.014) and delayed propulsion phase (p = 0.013) than the control group. Patients with moderate and severe AIS had asymmetrical gait periods in the left and right limbs (p<0.05). Significant differences in the center-of-pressure excursion index (CPEI) were found between the moderate and severe AIS and control groups (p = 0.003).

**Conclusion:**

Moderate and severe AIS significantly influenced walking performance; however, no significant differences were observed between adolescents with mild AIS and healthy controls. Thus, early intervention could target the prevention of specific functional deficits and prevent it from progressing to a severe state.

## Introduction

Adolescent idiopathic scoliosis (AIS) is the most common form of scoliosis prevalent in adolescents aged 10–18 years; it is accompanied by an unexplained spinal deformity with a Cobb angle of at least 10° [1,2]. The main symptoms of AIS are changes in body posture, such as unlevel shoulders, rib prominence, or waistline asymmetry [3,4]. Owing to spinal mobility and body posture changes, locomotion patterns may be altered during each step [5]. Unfortunately, the underlying mechanisms linking spinal deformity in individuals with AIS to foot characteristics and walking performance remain unclear.

To obtain detailed information about the underlying mechanism, a better understanding of the range of physical parameters in patients with AIS is essential, including foot morphology characteristics, plantar pressure distribution, and gait performance. Plantar pressure measurement and gait analysis are widely accepted as vital biomechanical parameters for quantitative assessment of human gait which can provide useful information on foot function and assist with the development of more effective preventive and interventional strategies [6–9]. Several studies have indicated that the center-of-pressure (COP) trajectory in patients with AIS is different from that in healthy controls [8,10–13]. Some previous studies have shown a difference in gait patterns between adolescents with untreated AIS and their healthy peers; conversely, other studies found no significant differences in gait analyses [5,14–17]. Therefore, some controversy remains in the existing literature. The magnitude of AIS is determined on the basis of the Cobb angle as follows: mild scoliosis, Cobb angle <20°; moderate scoliosis, Cobb angle of 20° to 45°; and severe scoliosis with a Cobb angle of >40° or 45° [1,18,19]. None of these studies have linked differences in plantar pressure distribution and gait to different severity levels of AIS, and the relationship between the severity levels of AIS and walking performance needs to be investigated.

Foot structure can significantly influence dynamic foot function, and the relationship between foot morphology and function has long been studied [20,21]. For example, medial displacement of the COP may be related to foot morphology with low arches [10]. Previous studies indicated that foot posture could affect the COP, particularly the propulsive stage of the stance phase during gait [22]. To our knowledge, most studies have not mentioned the difference in foot morphology between AIS and control groups and the impact of foot posture on walking performance in patients with AIS. Therefore, whether foot posture has significant effects on gait performance in individuals with AIS and the differences between patients with different levels of AIS and normal controls are unknown.

Based on the above considerations, the purpose of this study was to investigate the differences in plantar pressure distribution and gait in adolescents with mild, moderate, and severe scoliosis compared to controls while considering foot posture as a covariate.

## Materials and methods

### Registration

This study protocol was registered in the World Health Organization International Clinical Trials Registry Platform of the China Clinical Trial Register under registration No. ChiCTR2000033362.

### Ethics statement

This study was conducted in accordance with the principles of the Declaration of Helsinki and was approved by the ethics committee of the Affiliated Xuzhou Rehabilitation Hospital of Xuzhou Medical University (ethics code No. XKYL2020004). All participants and their legal guardians provided written informed consent before the start of the experiment.

### Participants

Sixty-four patients with AIS and 32 controls volunteered to participate in this study from April 2020 to October 2020 in China. Participants were recruited via offline and online systems concurrently. The participants were recruited from Xuzhou Central Hospital, the Affiliated Xuzhou Rehabilitation Hospital of Xuzhou Medical University, and some community hospitals, and included online platforms (WeChat, QQ, and Weibo), posters, pamphlets, and community advertisements. The main inclusion criteria were as follows: diagnosis of AIS via clinical and radiological methods by professional medical personnel; male and female patients aged 10–18 years who have agreed to undergo longitudinal plain radiography for Cobb angle measurements before the start of the experiment; patients with a left-lumbar and a right-thoracic component of their curves, of varying severity; limb length discrepancy within 1 cm; right limb dominance; no previous specific treatments for scoliosis; no congenital or preexisting musculoskeletal deformities, nervous system deformities, and infectious, traumatic, and psychiatric diseases; no disorder or surgery history of the spine, lower limbs, and feet; able to walk normally without assistance; and able to understand and follow instructions. The exclusion criteria were as follows: diagnosis of non-idiopathic scoliosis; age >18 years; had different deformities affecting normal locomotion; severe deformity of the lower limbs and feet; >1-cm limb length inequality; history of previous treatment for scoliosis; and inability to comprehend and follow task instructions.

### Measurement procedure

#### Clinical assessment

Demographic information of the participants was obtained via an inquiry and questionnaire. Weight and height were measured using an automatic weight/height measurement system. The Cobb angles on radiographs and ankle ranges of motion were assessed by the same experienced physiotherapist. Patients with AIS were classified as mild (Cobb angle < 20°), moderate (Cobb angle of 20° to 45°), and severe (Cobb angle > 45°) according to the major curve Cobb angles based on the clinical convention. The foot posture index (FPI) was used to measure foot posture, and an experienced investigator examined subjects’ feet manually [23]. The FPI is composed of six items, and each item is scored on a scale of −2, −1, 0, +1, and +2 (negative values for clear signs of supination, zero for clear signs of neutral, and positive values for clear signs of pronation). The six items were used to classify the position of the feet as follows: (i) palpation of the talar head, (ii) observation of the supramalleolar/inframalleolar curvature, (iii) inversion/eversion of the calcaneus, (iv) medial prominence of the talonavicular joint, (v) congruence of the medial arch, and (vi) abduction/adduction of the forefoot on the rearfoot [24]. The summed score ranged from −12 to +12, with a smaller negative value indicating a more supinated foot and a larger positive value indicating a more pronated foot. The reference standards were as follows: normal = 0 to +5, pronated = +6 to +9, highly pronated = +10 to +12, supinated = −1 to −4, and highly supinated = −5 to −12 [24]. For the measurement of walking speed, subjects were requested to walk on a 10-m flat surface at a self-selected comfortable speed, and the time was recorded [25].

#### Foot morphology assessment

Foot feature parameters were extracted using a laser foot scanner (LSR) system (Vismach Technology Ltd). During scanning, all measurements were performed barefoot in a relaxed standing position with the eyes looking straight forward. Both feet of each subject were scanned using three-dimensional imaging with the LSR 3D Foot Scan V2.5.7.6 software. The scanning system could provide different views of the feet, including the plantar, dorsal, medial, lateral, toe, and heel views. The foot parameters used in the study were foot length, foot width, heel width, arch length, 1–5 metatarsal width, and arch index (AI). The AI, which correlated with foot posture and morphology, and notably, with the navicular height, was measured from the subjects’ standing scans and calculated by dividing the length of the foot into three equal portions (minus the toes) and dividing the footprint in the middle third by the total footprint of all three regions [26]. The reference criteria were as follows: AI ≤ 0.21 indicating high arch, AI ≥ 0.26 suggesting a flat arch, and normal arch lies between 0.21 < AI < 0.26 [27].

#### Plantar pressure and gait assessment

**Fig 1:**
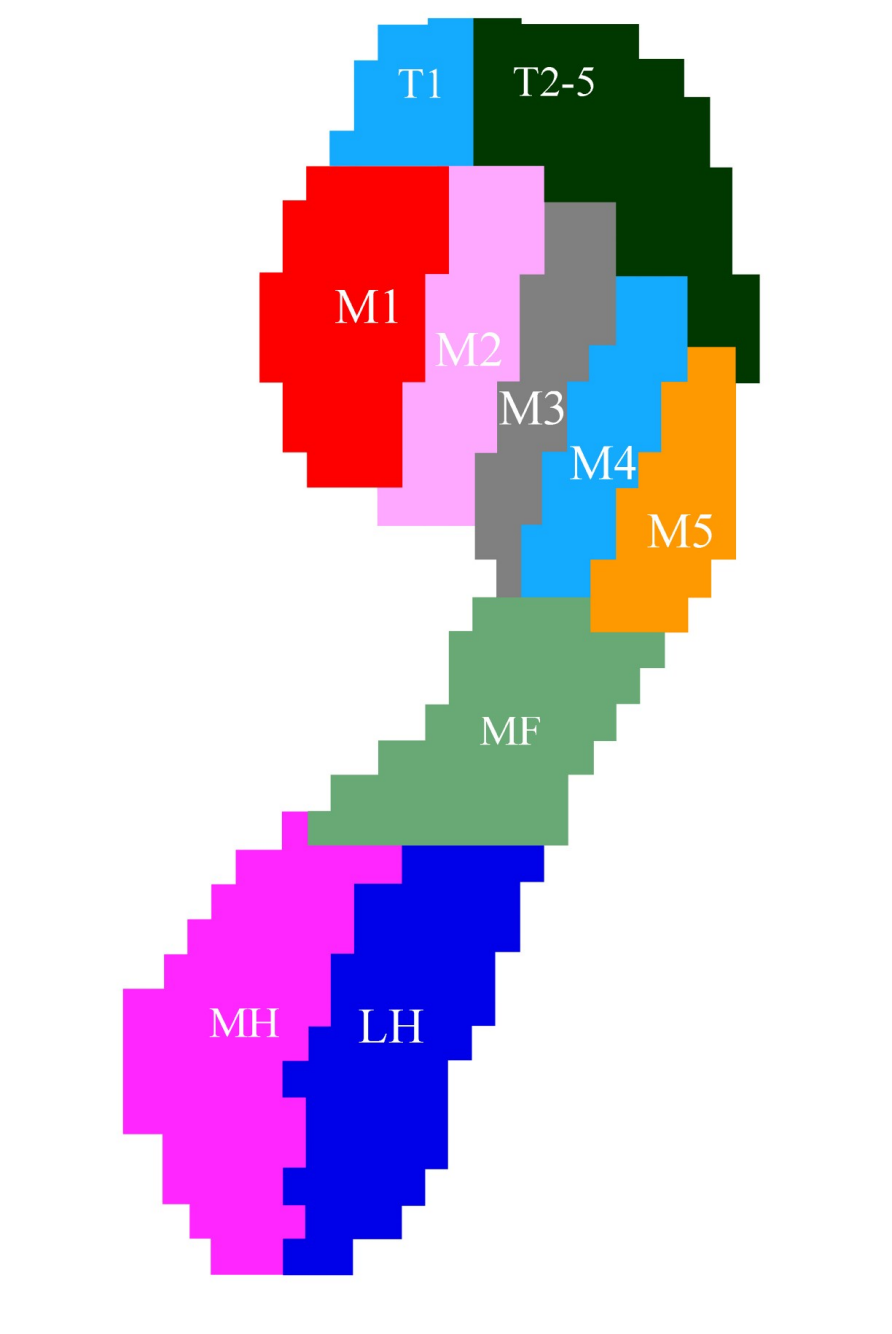
The divided regions of the foot. MH, medial heel; LH, lateral heel; MF, middle foot; M1-M5, 1st metatarsal to 5th metatarsal; T1, first big toe; T2-T5, 2nd toe to 5th toe.

The GaitScan pedal pressure system (Orthotic Group, Markham, Ontario, Canada), known to have high reliability and repeatability, has been previously demonstrated to capture plantar pressure and gait characteristics [28,29]. GaitScan is composed of a 578 × 418 × 12-mm force floor mat incorporating 4,096 pressure sensors, with a sampling frequency of approximately 125 Hz. Before the test, the participants were given several trials of walking on the platform to familiarize themselves with the test process. During the test, the participants walked barefoot at a self-selected comfortable speed with eyes looking straight ahead and performed an average of three trials, from which data were obtained for analysis. The two-step walking method was used in the data collection because it requires fewer trials and can effectively ensure that the participants are in contact with the pressure platform. In addition, the two-step method has been shown to be sufficient to achieve steady-state walking with equivalent reliability [22,30,31]. The parameters were chosen for analysis as follows: 1) seven foot regions were identified and the impulses beneath the feet were normalized by dividing the impulse for a given foot region by the total impulse of the foot in the rearfoot and forefoot, except for the midfoot region, yielding seven normalized impulse measures, namely (i) % medial heel for the rearfoot, (ii) % lateral heel for the rearfoot, (i) % 1st metatarsal, (ii) % 2nd metatarsal, (iii) % 3rd metatarsal, (iv) % 4th metatarsal, and (v) % 5th metatarsal for the forefoot. The divided regions of the foot are shown in **Fig 1**. 2) The start, end, and peak times of pressure under the medial heel, lateral heel, and 1–5 metatarsals. 3) The stance period during gait was divided into three phases as follows: (i) loading response (0 to 15% of stance), (ii) midstance phase (>15 to 65% of stance), and (iii) propulsion phase (means combination of terminal stance with pre-swing) (>65 to 100% of stance); the start time and end time of the gait cycle with the loading response, midstance, and propulsion phases were recorded [22]. 4) Foot function throughout the gait cycle was measured using the COP excursion index (CPEI). CPEI has previously demonstrated to be sensitive to changes in foot alignment and is defined as the excursion of the COP from a construction line drawn from the first and last points of each foot’s COP trajectory measured at the distal third of the foot, and the CPEI value is normalized by foot width [32]. 5) The medial-to-lateral rearfoot balance was calculated using the ratio of the medial heel impulse to the lateral heel impulse (M/L). 6) The T1/M1 ratio indicates the ratio of the impulse from the big toe to the impulse from the first metatarsal.

### Statistical analyses

Both feet of each participant were selected for the testing and analysis. When comparing the groups, only the left foot was used for the analysis. All statistical analyses were performed using IBM SPSS version 25.0 software (SPSS Inc., Chicago, IL, USA), and the significance level was set at p < 0.05. The Shapiro-Wilk test was used to evaluate the normality of distribution, and Levene’s test was used to test the equality of variance. The chi-square test was used for categorical variables, one-way analysis of variance for normally distributed continuous variables, and the nonparametric Kruskal-Wallis test was used for non-normally distributed variables. One-way analysis of variance with Bonferroni correction was performed, and a post hoc comparison of the mean values between the different groups was performed. For within-group comparisons, a paired sample *t-test* or Wilcoxon nonparametric test was used, where appropriate. The levels of AIS severity compared with healthy status were used as the grouping variable in the univariate analysis of covariance (UANCOVA) and CPEI, indicating that foot function was introduced as a dependent variable. Multiple analyses of covariance adjusted for age, sex, body mass index (BMI), FPI, AI, and walking speed were also performed.

## Results

### Participants’ characteristics

The study included 96 adolescents and 32 healthy peers in the control group and 64 patients with AIS in the AIS group, consisting of 18 patients with mild AIS (mean ± SD Cobb angle, 14.4° ± 3.5°), 32 with moderate AIS (30.6° ± 6.5°), and 14 with severe AIS (49.6° ± 3.4°). The mean ± SD age of the participants in the total sample was 13.29 ± 1.75 years, and no significant difference in age was found among the groups. The height of the mild AIS group was higher than that of the control group (p < 0.05); however, no statistically significant difference was found between the other groups. As shown in **Table 1**, when comparing the severe AIS group with the control group, statistically significant differences in weight and BMI were found, revealing that the subjects with severe AIS had lower body weights and BMIs. In the three AIS groups, the proportion of women was much higher than that of men, at 84.37% (54/64) and 15.63% (10/64), respectively. Sex-related differences were observed between the severe AIS and control groups.

**Table 1:** Characteristics of the participants with mild, moderate, and severe AIS as compared with those of the controls.

Parameters	Control	Mild AIS	Moderate AIS	Severe AIS
	(n = 32)	(n = 18)	(n = 32)	(n = 14)
Age (years)	13.13 (1.85)	13.28 (1.13)	13.69 (2.13)	12.86 (1.41)
Sex (woman/man)	22/10	14/4	26/6	14/0^**†**^
Height (cm)	161.91 (7.63)	166.78 (3.35)^**†**^	163.50 (6.50)	164.57 (4.80)
Weight (kg)	48.97 (8.27)	49.06 (6.37)	47.56 (8.25)	42.36 (3.08)^**†**^
BMI (kg/m^2^)	18.59 (2.41)	17.62 (2.12)	17.77 (2.90)	15.63 (2.41)^**†**^
Cobb angle (°)	/	14.4 (3.5)	30.6 (6.5)	49.6 (3.4)

Abbreviations: AIS, adolescent idiopathic scoliosis; /, not applicable.

^**†**^Significant difference compared to the control group (P < 0.05).

### Foot morphology and posture

**Fig 2:**
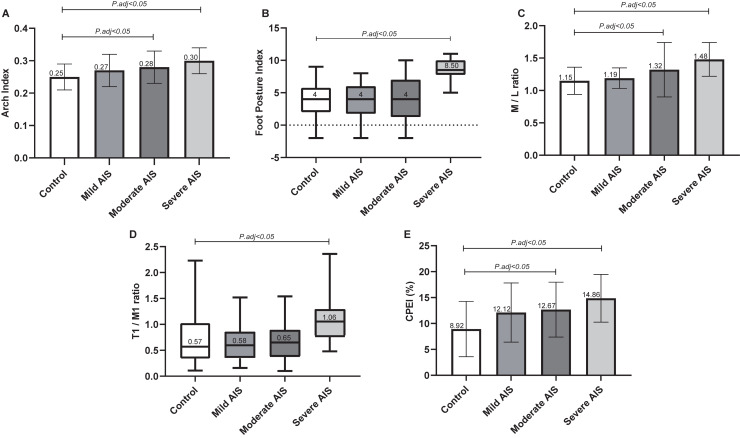
Results of between groups comparison with Bonferroni correction in Arch Index (A), Foot Posture Index (B), M/L ratio (C), T1/M1 ratio (D), and CPEI (E). M/L ratio, the ratio of the medial heel impulse to the lateral heel impulse; T1/M1 ratio, the ratio of the impulse from the big toe to the impulse from the first metatarsal; CPEI, the center of pressure excursion index; AIS, adolescent idiopathic scoliosis; P.adj, adjusted p-value.

In contrast to foot length, foot width, heel width, arch length, 1–5 metatarsal width, ankle dorsiflexion, and plantar flexion based on the three-dimensional scanner measurements showed no significant differences among the four groups. The results are presented in **Table 2**. AI was much higher in the moderate and severe AIS groups than in the control group (p< 0.05, **Fig 2**), indicating that patients in the moderate and severe AIS groups tended to have a lower arch than those in the control group. A significant difference was found in the FPI between the severe AIS and control groups (**Fig 2**), suggesting that individuals with severe AIS tended to have a more pronated foot posture.

**Table 2:** Comparison of foot posture and morphology between the mild, moderate, and severe AIS and control groups.

Parameters	Control	Mild AIS	Moderate AIS	Severe AIS	ANOVA/K-W p
	(n = 32)	(n = 18)	(n = 32)	(n = 14)	
Foot length (mm)	246.06 (15.34)	237.06 (12.25)	241.91 (15.54)	239.79 (13.20)	0.190
Foot width (mm)	91.31 (5.42)	88.94 (5.50)	91.78 (6.74)	91.07 (5.82)	0.432
Heel width (mm)	60.97 (4.35)	60.56 (3.68)	60.22 (5.28)	60.07 (4.07)	0.898
Arch length (mm)	177.94 (11.00)	171.61 (8.64)	174.94 (11.43)	173.57 (8.83)	0.206
1–5 metatarsal width (mm)	65.47 (4.94)	64.56 (5.50)	64.69 (4.31)	65.07 (3.32)	0.888
Arch index	0.25 (0.04)	0.27 (0.05)	0.28 (0.05)†	0.30 (0.04)†	**0.018**
Foot posture index	4 (2, 5.75)	4 (1.75, 6)	4 (1.25, 7)	8.50 (7.75, 10)†	**<0.001**
Ankle dorsiflexion (°)	13 (3.46)	14.44 (3.55)	14.47 (3.97)	13 (3.69)	0.293
Ankle plantarflexion (°)	40.69 (7.77)	42.89 (4.10)	40.28 (5.54)	37.43 (5.32)	0.106

Abbreviations: AIS, adolescent idiopathic scoliosis; ANOVA, one-way analysis of variance; K-W, Kruskal-Wallis rank test.

^**†**^Significant difference compared to the control group (P < 0.05).

### Gait analysis

**Fig 3:**
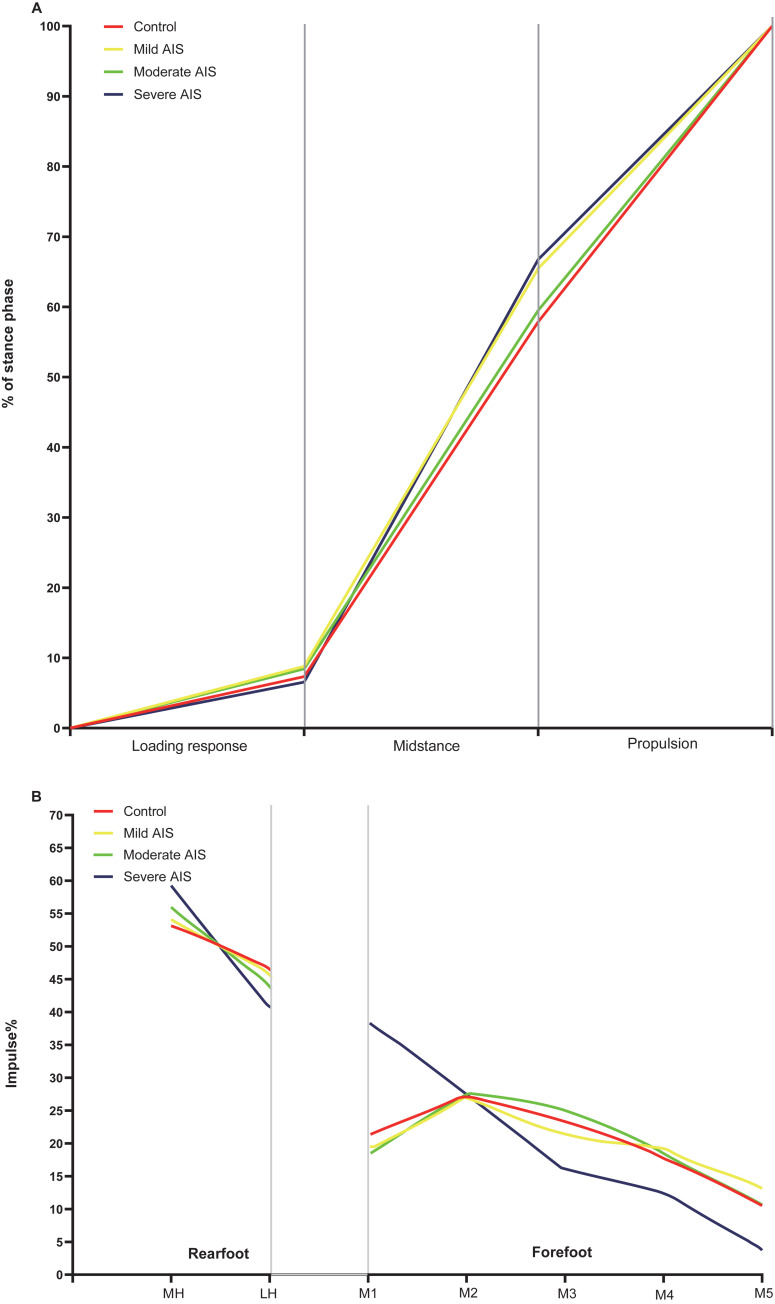
Results in gait (A) and plantar pressure distribution (B) between groups comparison. MH, medial heel; LH, lateral heel; M1-M5, 1st metatarsal to 5th metatarsal; (MH+LH) impulse% = 100%; (M1+M2+M3+M4+M5) impulse% = 100%.

The severe AIS group had advanced and longer midstance, and a delayed propulsion phase compared with the control group (p < 0.05, **Table 3**, **Fig 3)**. Gait parameters such as the loading response, midstance, and propulsion phases were not found to be different between the left and right in the groups **(Table 3)**. However, the moderate and severe AIS groups had asymmetrical gait periods in both the left and right limbs (p < 0.05), revealing that the patients in the moderate and severe AIS groups might have had abnormal asymmetrical gait patterns. Regarding walking speed, the moderate and severe AIS groups had slower walking speeds than the control group **(Table 4)**.

**Table 3:** Gait analysis for comparison between the mild, moderate, and severe AIS and control groups.

			Gait cycle (%)			Within-group P		
			Loading response	Midstance	Propulsion	Loading response	Midstance	Propulsion
Control	Left	Start	0	7.33 (3.40)	57.87 (10.68)	0.771	0.694	0.450
		End	7.33 (3.40)	57.87 (10.68)	100			
		Total	7.33 (3.40)	50.56 (10.59)	42.13 (10.68)			
	Right	Start	0	7.18 (3.87)	59.23 (9.56)			
		End	7.18 (3.87)	59.23 (9.56)	100			
		Total	7.18 (3.87)	51.12 (10.64)	40.77 (9.56)			
Mild AIS	Left	Start	0	8.79 (3.65)	65.46 (11.22)	0.689	0.566	0.486
		End	8.79 (3.65)	65.46 (11.22)	100			
		Total	8.79 (3.65)	56.64 (11.31)	34.54 (11.22)			
	Right	Start	0	8.39 (4.37)	63.35 (12.79)			
		End	8.39 (4.37)	63.35 (12.79)	100			
		Total	8.39 (4.37)	54.93 (14.00)	36.65 (12.79)			
Moderate AIS	Left	Start	0	8.44 (4.23)	59.50 (9.23)	0.244	0.323	0.673
		End	8.44 (4.23)	59.50 (9.20)	100			
		Total	8.44 (4.23)	51.05 (10.60)	40.50 (9.20)			
	Right	Start	0	7.46 (3.68)	60.17 (9.64)			
		End	7.46 (3.68)	60.17 (9.64)	100			
		Total	7.46 (3.68)	52.70 (9.49)	39.83 (9.64)			
Severe AIS	Left	Start	0	6.56 (3.00)†	66.76 (10.83)†	0.169	0.942	0.747
		End	6.56 (3.00)	66.76 (10.83)†	100			
		Total	6.56 (3.00)	60.19 (9.78)†	33.24 (10.83)†			
	Right	Start	0	5.62 (2.22)	65.96 (5.15)			
		End	5.62 (2.22)	65.96 (5.15)	100			
		Total	5.62 (2.22)	60.35 (6.09)	34.04 (5.15)			
ANOVA P			0.237	**0.014**	**0.013**			

Abbreviations: AIS, adolescent idiopathic scoliosis; ANOVA, one-way analysis of variance; within-group, within-group comparison.

^**†**^Significant difference compared to the control group (P < 0.05).

**Table 4:** Comparison of plantar pressure distributions between the mild, moderate, and severe AIS and control groups.

											ANOVA/K-W p
			Control		Mild AIS		Moderate AIS		Severe AIS		
			Left	Right	Left	Right	Left	Right	Left	Right	
Rearfoot	Impulse %	Medial heel	53.12 (4.15)	53.12 (6.28)	54.09 (3.24)	53.70 (3.84)	55.97 (5.79)^**†**^	55.74 (6.40)	59.27 (4.40)^**†**^	61.17 (5.39)	**0.001**
		Lateral heel	46.87 (4.15)	46.88 (6.28)	45.91 (3.24)	46.30 (3.84)	44.03 (5.79)^**†**^	44.26 (6.40)	40.73 (4.40)^**†**^	38.83 (5.39)	**0.001**
		M/L ratio	1.15 (0.21)	1.17 (0.31)	1.19 (0.16)	1.17 (0.18)	1.32 (0.42)^**†**^	1.31 (0.34)	1.48 (0.26)^**†**^	1.63 (0.40)	**0.004**
Forefoot		1st Metatarsal	21.22 (12.80)	20.69 (10.56)	19.36 (7.87)	21.46 (10.31)	18.22 (11.01)	21.23 (12.33)	38.49 (9.42)^**†**^	33.94 (12.11)	**<0.001**
		2nd Metatarsal	27.29 (5.42)	26.14 (5.58)	27.02 (4.27)	26.08 (7.30)	27.60 (6.11)	28.61 (5.65)	27.62 (4.84)	27.61 (6.73)	0.982
		3rd Metatarsal	23.48 (5.84)	24.74 (5.17)	21.44 (6.73)	21.52 (7.03)	25.22 (4.85)	23.29 (5.19)	16.09 (7.81)^**†**^	21.79 (6.76)	**<0.001**
		4th Metatarsal	17.52 (5.70)	18.43 (5.75)	19.07 (5.79)	17.89 (7.95)	18.31 (4.87)	17.31 (4.83)	12.24 (4.44)^**†**^	12.84 (3.60)	**0.002**
		5th Metatarsal	10.51 (7.06)	10 (5.76)	13.13 (8.96)	13.04 (6.85)	10.65 (7.42)	9.57 (4.54)	3.73 (0.98)^**†**^	3.86 (1.94)	**0.003**
		T1/M1 ratio	0.57 (0.34, 1.03)	0.63 (0.42, 1.00)	0.58 (0.35, 0.85)	0.65 (0.38, 0.98)	0.65 (0.38, 0.89)	0.65 (0.35, 1.16)	1.06 (0.76, 1.29)^**†**^	1.06 (0.66, 1.35)	**0.012**
		CPEI (%)	8.92 (5.33)	9.99 (4.74)	12.12 (5.71)	11.06 (6.58)	12.67 (5.29)^**†**^	12.25 (6.61)	14.86 (4.60)^**†**^	14.08 (3.06)	**0.003**
		Walking speed (m/s)	1.19 (0.05)		1.21 (0.04)		1.17 (0.06)^**††**^		1.17 (0.05)^**††**^		**0.038**
		Gait period (s)	0.81 (0.09)	0.81 (0.10)	0.81 (0.05)	0.82 (0.05)	0.81 (0.10)	0.79 (0.08)	0.85 (0.05)	0.78 (0.06)	0.550
Within-group p		Medial heel	0.993		0.734		0.880		0.220		
		Lateral heel	0.993		0.734		0.880		0.220		
		M/L ratio	0.754		0.788		0.872		0.177		
		1st Metatarsal	0.786		0.469		0.182		0.322		
		2nd Metatarsal	0.257		0.628		0.382		0.995		
		3rd Metatarsal	0.151		0.970		0.079		**0.040**		
		4th Metatarsal	0.352		0.574		0.454		0.696		
		5th Metatarsal	0.653		0.970		0.391		0.826		
		T1/M1 ratio	0.100		0.413		0.151		0.848		
		CPEI (%)	0.341		0.416		0.799		0.388		
		Walking speed (m/s)	/		/		/		/		
		Gait period (s)	0.743		0.246		**0.025**		**0.001**		

Abbreviations: AIS, adolescent idiopathic scoliosis; ANOVA, one-way analysis of variance; K-W, Kruskal-Wallis rank test; within-group, within-group comparison.

/, not applicable.

^**†**^Significant difference compared to the control group (P < 0.05).

^**††**^Significant difference compared with mild AIS group (p < 0.05).

### Plantar pressure distribution

In the rearfoot, the percentages of impulse in the medial and lateral heels were significantly higher and lower, respectively, in adolescents with moderate and severe AIS than in their healthy peers (p < 0.05). The medial-to-lateral rearfoot balance (M/L ratio) was significantly different between the moderate and severe AIS and control groups (p < 0.05), being approximately 1.15, 1.32, and 1.48 in the moderate and severe AIS groups, respectively, indicating that impulses were imbalanced across the medial and lateral rearfoot sides. In the forefoot, the percentage of impulses in the first metatarsal was significantly higher in the severe AIS group than in the control group, and the percentages of impulses in the third, fourth, and fifth metatarsals were significantly lower in the severe AIS group than in the control group (p < 0.05). The results demonstrated that the ratio of the impulse from the big toe to the impulse from the first metatarsal (T1/M1 ratio) was significantly higher in the severe AIS group than in the control group (p < 0.05). In terms of the CPEI, significant differences were found between the moderate and severe AIS and control groups (p < 0.05). Univariate analysis of covariance revealed significant differences in CPEI between the groups after adjusting for age, sex, BMI, FPI, AI, and walking speed (UANCOVA F = 2.68, p = 0.009). No significant plantar pressure difference was found between the mild AIS and control groups. In the intra-group comparison, only the percentage of impulses in the third metatarsal was found to be asymmetrical in the severe AIS group. All of the above-mentioned results are shown in **Table 4** and **Fig 3**.

## Discussion

The present study had two purposes. First, this study aimed to compare the plantar pressure distribution, kinematic parameters of gait, foot morphology, and posture between adolescents with mild, moderate, and severe AIS and their matched healthy peers. Second, the foot function of patients with AIS was examined while considering foot posture as a covariate. From the above-mentioned analysis, these following conclusions can be drawn: there were no significant differences between adolescents with AIS (even severe AIS) and their healthy peers in terms of foot length, foot width, heel width, arch length, 1–5 metatarsal width, ankle dorsiflexion, and plantar flexion; however, adolescents with moderate to severe AIS tended to have low foot arches, and those with severe AIS tended to have a more pronated foot posture than the controls. Some abnormal and asymmetrical gait patterns were found in the moderate and severe AIS groups, while these measures were similar between patients with mild AIS and controls. Plantar impulses were significantly imbalanced in adolescents with moderate and severe AIS. In terms of the CPEI, significant differences were found between the AIS particularly moderate to severe cases and control groups after adjusting for age, sex, BMI, FPI, AI, and walking speed.

Consistent with the reports of previous studies, female subjects have greater predisposition to AIS [33,34]. In the AIS group, the proportion of women (84.37%) was much higher than that of men (15.63%). Idiopathic scoliosis is a deformity without clear etiology. Studies have shown that the disease is likely to be related to genetic factors, growth imbalance, and estrogen. Severe scoliosis cases had lower body weights and BMIs than other cases, which indicated that scoliosis, particularly severe scoliosis, might influence the growth of the body in these adolescents [35,36]. Severe scoliosis cases might have shorter body height due to the presence of larger curvatures; however, no such differences were observed, probably due to the small sample size of severe cases. Many previous studies have reported that plantar pressure and gait are affected by several factors such as age, sex, BMI, and walking speed [37–39]. Therefore, plantar pressure distribution and gait in adolescents with mild, moderate, and severe scoliosis were analyzed and compared with matched healthy peers, while considering age, sex, BMI, and walking speed as covariates. After adjusting for these covariates, significant differences in foot function were found between the AIS and control groups.

In recent years, researchers have not only focused on the three-dimensional deformity of the spine, but have also focused on different body parts. With the development of medical biomechanics and the overall view of the body structure, studies have shown that biomechanical factors of the pelvis, lower limbs, and feet also have an impact on the formation of scoliosis. The foot structure could significantly influence dynamic foot function, which has been reported previously [40,41]. In a systematic review that investigated the relationship between foot posture and plantar pressure during walking, the authors concluded that plantar pressure characteristics differed according to foot posture [42]. To the best of our knowledge, most studies have not mentioned the difference in foot posture between AIS and control groups and the impact of foot posture on walking performance in these patients, which was investigated in this study. In fact, no significant differences in foot length, foot width, heel width, arch length, 1–5 metatarsal width, ankle dorsiflexion, and plantar flexion were reported between patients with AIS and healthy controls. However, patients with moderate to severe AIS tended to have low foot arches, and those with severe AIS tended to have a more pronated foot posture than the controls. There was major concern that these differences could affect the results during walking performance assessment; thus, a univariate analysis of covariance test was conducted, adjusting for the FPI and AI. However, significant differences in CPEI between the groups were still observed. Most of the patients in our study possessed a left-lumbar and a right-thoracic component of their curves for the spine; however, there still were 8 from both the groups in 64 cases having thoracic curve or lumbar curve with Cobb angle 0°, 2 cases in the mild AIS group, 5 cases in the moderate AIS group, 1 case in the severe AIS group. Therefore, this study was limited by the sample size and any differences between the side of scoliosis (e.g., right thoracic curve) to the corresponding (right) foot and contralateral (left) foot were not investigated. The CPEI is a measure of foot function and has been shown to be useful for evaluating foot posture [32,43]. In agreement with previous studies, it was observed that the more laterally deviated the COP in the cavus feet, the larger the area of the total COP excursion; however, the more medially deviated the COP in the planus feet, the smaller the area of the total COP excursion [20,42]. In this study, adolescents with moderate and severe AIS with flatter and pronated feet tended to have higher CPEI values, differing from previous studies that reported a smaller CPEI in the planus foot without scoliosis. In addition, the value of CPEI in healthy adolescents was much smaller than that reported in other literatures probably because this observational study had few cases and case selection bias, and adolescents were still in growing stages without fully developed feet. The structure of the spine axis muscle is both a receptor and an effector, which plays an important role in static or dynamic balance. However, the structure of AIS patients, particularly those with severe disease, is damaged, and patients may have balance disorders and be at risk of falling. Some studies have reported that patients with AIS have poor postural balance and a higher sway area due to dysfunction in various equilibrium factors [12]. In other words, the CPEI of patients with AIS might be affected not only by foot posture but also by scoliosis. This study found that the CPEI of patients with moderate and severe AIS was significantly higher than that of healthy controls; therefore, these patients compensate for postural asymmetry caused by changes in the shape of the spine through the vestibule and somatosensory system, such as the ankle proprioceptive system and increased energy consumption, which helps them maintain a stable posture while walking. When patients with AIS stand quietly and steadily, their balance is similar to that of healthy people; however, when walking, vision and body proprioception are challenged, and AIS patients often show some abnormalities, such as increased body swing. The position of the COP is abnormal, and the movement range of the COP is enlarged in the side directions. The abnormal lateral swing of the COP of AIS patients may be caused by an unbalanced torque when the body’s center of gravity moves laterally due to spinal deformity. Patients with AIS during walking with unbalanced plantar pressure distribution can also experience changes in the mutual positional relationship between body segments and aggravate their spinal deformities. In addition, the relative posture of the patient’s head, shoulders, spine, pelvis, legs, and feet on a three-dimensional plane can also change, further affecting the information input of balance receptors, thereby aggravating balance dysfunction. Whether abnormal plantar pressure distribution is one of the causes of AIS or the result remains to be further studied.

This study found that the midstance phase duration was increased and advanced, and the propulsion phase was delayed in patients with severe AIS as compared with their healthy peers. One possible explanation for the prolonged midstance phase was that severe AIS took more time to maintain a steady posture and prepared for the forefoot generating full force to thrust against the ground. The forefoot is known to be the only structure in contact with the ground during the terminal stance [44]. Thus, sufficient force must be generated over the forefoot to drive the lower limb and body forward [22,45]. The propulsion phase was delayed in patients with severe AIS, which revealed that these patients might have decreased the ability to facilitate forward propulsion of the body. The ratio of the impulse from the big toe to the impulse from the first metatarsal (T1/M1) was also calculated, an important calculation that could determine a trend if a patient with AIS had a hypermobile first ray or limited hallux range of motion. The results showed that the severe AIS group had much higher T1/M1 ratios than the controls and might have a tendency to develop a limited hallux range of motion or even hallux rigidity in the future. Consistent with these results, it was found that the percentage of impulses in the first metatarsal of the severe AIS group was much higher than that of the controls. In the rearfoot, the percentage of impulse in the medial heel was significantly higher than that in the lateral heel in adolescents with moderate and severe AIS than in their healthy peers, indicating that impulses were imbalanced across the medial and lateral rearfoot sides. In addition, for within-group comparison, the patients with moderate and severe AIS had asymmetrical gait periods in both the left and right limbs, indicative of the probable abnormal asymmetrical gait patterns in patients in the moderate and severe AIS groups. From these results, it is evident that plantar loading patterns differ between mild, moderate, and severe AIS. The results of this study could boost further research and provide a guide for using designated foot orthoses, insoles, and footwear for changing foot loading combined with conventional braces for the conservative treatment of AIS, and provide useful information on foot function and assist with the development of more effective preventive and interventional strategies.

The present study is novel as consideration given to the impact of the different levels of AIS severity since structural deformity might not influence function until the disease has progressed to a severe state [46]. Consistent with these results, no significant differences were found between patients with mild AIS and controls. In addition, foot posture, which could affect the COP, particularly the propulsive phase of stance during gait, was also investigated in patients with AIS in this study. However, this study has methodological considerations and limitations that should be stated herein. First, it had a cross-sectional design, and causal connections could not be inferred from the results. Second, it was difficult to draw conclusions about the influences of foot posture and scoliosis on the entire foot function because of device limitations such as the absence of associated data included electromyography data and kinetic parameters of gait. Finally, this study distinguished the magnitude of AIS determined on the basis of the Cobb angle, lacking consideration for sagittal radiography.

## Conclusions

Moderate to severe AIS significantly influenced walking performance; however, no significant differences were observed between adolescents with mild AIS and the controls. These results suggested that early intervention could prevent specific functional deficits such as poor body balance and avert it from progressing to a severe state. Future studies investigating the walking performance of patients with AIS should include foot posture as a concomitant factor not only for scoliosis, as foot posture is likely to influence functional performance. The use of the designated foot orthoses, insoles, and footwear for changing foot loading combined with conventional braces for the conservative treatment of AIS may improve foot function and assist with the development of more effective preventive and interventional strategies.

## Supporting information

S1 Table STROBE checklist. (DOCX)
